# Fecal Sample Collection for Gut Microbiome Research in a Prospective Cohort: A Pilot Study within the Australian Breakthrough Cancer Study

**DOI:** 10.1158/2767-9764.CRC-25-0445

**Published:** 2026-01-09

**Authors:** Simon Cheah, Jared Burke, Fiona J. Bruinsma, Melanie Evans, Helen Tsimiklis, Allison M. Hodge, Brigid M. Lynch, Graham G. Giles, Rashmi Sinha, Melissa C. Southey, Roger L. Milne

**Affiliations:** 1 Cancer Epidemiology Division, Cancer Council Victoria, Melbourne, Australia.; 2 Melbourne School of Population and Global Health, The University of Melbourne, Melbourne, Australia.; 3 Precision Medicine, School of Clinical Sciences at Monash Health, Monash University, Melbourne, Australia.; 4 Burnet Institute, Melbourne, Australia.; 5 Division of Cancer Epidemiology and Genetics, National Cancer Institute, Bethesda, Maryland.

## Abstract

Large prospective analyses of human gut microbiome profiles are needed to elucidate the role of microbiome variation in the development of disease. We conducted a pilot study to assess the feasibility of home fecal sample collection within a cohort study. A subset of cohort study participants was randomly selected and randomized into four groups defined by fecal sample collection method and questionnaire components. Of 1,093 invited participants, 610 (56%) opted-in, and of those, 88% returned a sample. Of those asked to provide a fecal sample via fecal occult blood test (FOBT) card and complete a short “day-of-sample” questionnaire, 49% returned a sample. Sample return was comparable for participants additionally asked to provide a sample via ethanol tube (51%), complete a food frequency questionnaire (48%), or complete both additional activities (49%). Whole-genome sequencing and metagenomic analysis on paired FOBT and ethanol samples showed that both collection methods provided sufficient quality and quantity of DNA for downstream metagenomic analyses and displayed highly concordant microbiome profiles. Home fecal sample collection for microbiome analysis is feasible in a large prospective cohort. Including additional components did not reduce the likelihood of participants completing all requested items.

**Significance:**

The expansion of this successful pilot to the larger Australian Breakthrough Cancer Study will facilitate future metagenomic and other host- and microbiome-related analyses in this large prospective cohort and potentially as part of an extended international pooling project.

## Introduction

The human microbiome is the ecological community of microorganisms (bacteria, archaea, fungi, protists, and viruses) and their metabolic products that inhabit the human body. The recent emergence of high-throughput DNA sequencing technologies has enabled a rapid expansion of culture-independent research into the association between the human microbiome and health and disease (1, 2). The collection and storage of host and microbiota samples now, for use in future multiomic analyses, could benefit the rapidly advancing field of molecular epidemiology (3). Such analyses might be usefully integrated with prospectively collected data on variables such as diet and medical history, allowing the investigation of the temporality, direction, and causality of associations between microbiome variation and health outcomes in prospective cohorts.

The gut microbiome can be reliably measured in fecal samples, but collection methods vary and have different considerations with regard to sample preparation, collection, storage, study coordination time, participant burden, and costs (4, 5). Fecal occult blood test (FOBT) cards have been demonstrated to be an efficacious and cost-effective means of collecting fecal samples for microbiome analysis (6–8). Another cost-effective method is collecting a small piece of feces in a tube containing 95% ethanol (4, 9, 10). Ideally, a cohort study would collect samples by both FOBT and ethanol, as FOBT samples may perform better for metagenomic analysis, whereas samples collected in 95% ethanol might perform better for metabolomic analyses (5, 8, 11). However, it is unclear whether this represents an excessive burden on study participants.

The Australian Breakthrough Cancer (ABC) Study is cohort of 56,282 Australians ages 40 to 74 years at recruitment in 2014 to 2018. The study aims to investigate the role that our genes, lifestyle, and environment play in the development of cancer and other diseases. Participants were recruited primarily through advertising on social media (12). At recruitment, participants gave consent, completed epidemiologic questionnaires, and provided a saliva sample, from which DNA was extracted and stored. Approximately 18% of participants also provided a blood sample, from which plasma and DNA were extracted and stored, along with aliquots of the remaining blood. The first wave of follow-up by online questionnaire was conducted in 2019 to 2021, inviting roughly a third of participants each calendar year by the order in which they were recruited. It is planned to go back to participants every 3 years, on a rolling basis, to invite them to complete further questionnaires and contribute additional biological samples.

It is anticipated that many of the aims of the ABC Study will be met by pooling its data with those from other cohort studies from around the world. This approach will enable the generation of evidence based on adequate sample sizes to reach definitive conclusions about the associations of potential risk factors with the development of cancer, particularly for less common cancers, less common exposures, and exposures with high within-person variability, such as the gut microbiome (13, 14). Studies measuring the temporal stability of the microbiome profile suggest that large case and participant sample sizes will be necessary to detect disease associations (15, 16). For data pooling to be feasible, contemporary studies will need to collect and store samples from “healthy” participants (i.e., prior to a cancer diagnosis) in a way that is scalable (across many thousands of study participants), feasible (in terms of both logistics and cost), and comparable across different cohort studies internationally (2, 9).

In addition, because diet can affect the microbiome profile, it is of interest to collect dietary data at the time of fecal sample collection (17, 18). However, dietary questionnaires tend to be lengthy, the burden of which could represent a further barrier to participation in sample collection. Thus, we aimed to carry out a pilot study to assess the feasibility of self-collection of fecal specimens from participants in the ABC Study, including the effect on participation of (i) requesting a sample in ethanol, in addition to FOBT cards, and (ii) additionally requesting the contemporaneous completion of a food frequency questionnaire (FFQ).

## Materials and Methods

Eligible ABC Study participants were those who had provided an email address and were recruited to the study in the latter half of 2016 (to avoid overlap with the first wave of follow-up). Between September and December 2019, 1,093 eligible participants were randomly selected, using weights to ensure approximately equal numbers of males and females, and invited to participate in the ABC Gut Pilot Study (Supplementary Figs. S1–S3). Prior to invitation, participants were randomized into one of four groups defined by different combinations of fecal sampling methods and questionnaires to be completed, as outline in Table 1.

**Table 1.** Study groups to which pilot study participants were randomized.

Group	Sample(s)	Questionnaire(s)
A	FOBT cards only	dosQ
B	FOBT cards + 95% ethanol tube	dosQ
C	FOBT cards only	dosQ + FFQ
D	FOBT cards + 95% ethanol tube	dosQ + FFQ

All participants (groups A, B, C, and D) opting-in were asked to give a fecal sample using FOBT cards. This required them to smear a small amount of feces in two windows on each of two cards (four smears in total). They were also asked to complete an online questionnaire on the day they produced the sample [day-of-sample questionnaire (dosQ)]. The questionnaire included items considered relevant for microbiome variation such as drug use (antibiotics, bile production medications, and acid reducers) over last 2 months; body size (height and weight); fecal consistency; consumption of probiotics/prebiotics and fermented foods; cigarette smoking; and recent illness or hospitalization.

Half of the participants (groups B and D) were randomized to provide an additional fecal sample in a tube containing 2.5 mL of 95% ethanol.

Half of the participants (groups C and D) were randomized to also complete a 137 item FFQ, consisting of questions about usual eating and drinking habits over the previous 12 months. This questionnaire, adapted from an earlier FFQ developed by Cancer Council Victoria (CCV) and validated in a substudy of the Melbourne Collaborative Cohort Study (19), was also completed at entry into the ABC Study.

An information sheet specific to each group was provided to all invited participants. This specified what types of samples would be requested and which questionnaires should be completed, with an estimated questionnaire completion time of 10 to 15 minutes for groups A and B and of 15 to 30 minutes for groups C and D.

We contacted 110 of the invited participants by phone to obtain feedback and assess barriers to completion, an email was sent if they were not contactable by phone. Some were participants who agreed to participate but did not complete the study (*n* = 73), and others were those who did not respond to the invitation (*n* = 37).

Fecal sampling kits were prepared by CCV (Supplementary Figs. S4–S6). All groups received a reply-paid addressed return envelope, letter with sample collection instructions, gloves, applicators, toilet liners, two barcoded FOBT cards, and foil envelope. Groups C and D additionally received a barcoded 2.5 mL ethanol-containing PET tube with a barcoded zip lock bag. FOBT cards were approximately 12 cm by 6 cm when unfolded, with two marked rectangular areas for sample collection, fields for participants to note the time and date of sample collection, as well as written instructions on how to collect the sample.

Participants returned the samples directly to the Precision Medicine Biorepository (PMB), Monash University. On receipt, the collection time and date (as noted by the participant), receipt date, and any relevant notes (e.g., low volume) were recorded. All samples were stored at −80°C.

Trial DNA extraction was conducted for samples on FOBT cards and in ethanol tubes collected from four participants (two FOBT card samples per participant) received 3 to 10 days after sample collection and from a fecal sample that had been frozen at −20°C within an hour of collection (control sample). Details about the participants included in the trial extraction are summarized in Supplementary Table S1. This includes information about the state of residence and time the sample was in transit (samples collected in states such as Western Australia and Queensland may have been exposed to higher temperatures and longer transit times than samples collected and transported in Victoria); these factors may have an effect on the species and quality of DNA.

All participants gave written informed consent. The study was conducted in accordance with the Declaration of Helsinki and approved by CCV’s Human Research Ethics Committee (1403).

### Metagenomic sequencing

DNA was extracted from the 95% ethanol fecal sample and two areas of the FOBT card for three ABC participants using the Qiagen QIAamp PowerFecal Pro DNA Kit (nine DNA samples in total). Metagenomic shotgun sequencing was conducted at Genewiz on the Illumina HiSeq platform in a 2 × 150 bp parried-end configuration generating a minimum of 10G of data per sample.

Sequencing data of paired FOBT and ethanol samples from the three ABC participants were analyzed using the Real Time Genomics (RTG) metagenomic composition pipeline (v3.12.1) consisting of read filtering (removal of poor-quality and human-mapping reads), alignment to a set of known bacterial and viral reference genomes (non-eukaryota subset of Genomes Online GOLD genomes and NCBI virus genomes available with RTG tools), and calculation of species abundance, diversity, and composition metrics.

## Results

### Responses to gut microbiome pilot study invitation

Of the 1,093 eligible participants invited to participate in the pilot study, one had moved overseas and was therefore excluded. Of those remaining, 610 (56%) opted-in, 57 (5.2%) refused, and 426 (39%) did not respond to the invitation. Participant responses to the initial invitation to participate in the pilot study and participant completion of study components, by study group, are summarized in Table 2. Participants asked to complete the FFQ (groups C and D) were more likely to refuse (6.6%) than those not asked to complete the FFQ [groups A and B, 3.8%, Fisher exact test *P* value (P_F-exact_) = 0.042]. There was no apparent pattern related to the inclusion of additional study components (request for fecal sample in 95% ethanol or the request to complete an FFQ) in the proportion of participants opting-in.

**Table 2.** Response to invitations and completion of pilot study components.

​	Group A	Group B	Group C	Group D
	FOBT cards	FOBT cards	FOBT cards	FOBT cards
	dosQ	95% ethanol dosQ	dosQ FFQ	95% ethanol dosQ FFQ
	*N* (%)^a^	*N* (%)^a^	*N* (%)^a^	*N* (%)^a^
Invited and eligible	274	273	273	273
Refused	11 (4.0%)	10 (3.6%)	17 (6.2%)	19 (7.0%)
No response	109 (40%)	103 (38%)	115 (42%)	99 (36%)
Opted-in	154 (56%)	160 (59%)	141 (52%)	155 (57%)
FOBT^b^ sample	135 (49%)	139 (51%)	130 (48%)	134 (49%)
FOBT^b^ + 95% ethanol sample	​	139 (51%)	​	133 (49%)
Fecal^c^ sample + dosQ	116 (42%)	120 (44%)	115 (42%)	113 (41%)
Fecal^c^ sample + dosQ + FFQ	​	​	109 (40%)	110 (40%)

a The denominator for all percentage figures is the total number of “invited and eligible” participants for that group.

b Returned at least one of the two FOBT cards (all except six of these returned both cards).

c Returned at least one FOBT card or a 95% ethanol sample (all except one returned both FOBT cards).

Overall, 49% of invited participants (88% of those who opted-in) returned an FOBT fecal sample, with very little difference in this percentage between the four groups (49%, 51%, 48%, and 49% for groups A, B, C, and D, respectively); nearly all of those also returned a fecal sample in 95% ethanol, when asked (Table 2). Overall, 86% of participants that returned a fecal sample also completed the dosQ, but this did not vary substantially between the four study groups (P_F-exact_ = 0.34).

### Participant feedback

Participants were asked within the dosQ to note and describe any problems or concerns they had with the sample collection process. The most common concern expressed by participants (*n* = 11) was that they were unclear about the ideal timing of sample collection, including whether to take each of the samples on the same or different days. Other concerns included issues logging in to the study web portal (*n* = 9) and frustration with being unable to skip the sample time/date question if they could not remember (*n* = 1).

We attempted to contact 37 nonresponders, and 23 (62%) provided feedback. Of these, 39% responded that they did not see the invitation to participate in the pilot study, whereas others indicated that they did not want to participate (26%) or that it was a bad time for them (26%).

A total of 60 (83%) of 73 noncompleters provided feedback. A majority of these (53%) did not realize there was a questionnaire associated with this aspect of the study. Another subset of participants (27%) indicated that they had either forgotten to complete the questionnaire or did not realize they had not completed the questionnaire. Others indicated that they were too busy or that it was a bad time for them (8%).

### Analysis of samples

The average time from participant sample collection to receipt at PMB was 5.4 days, with a median of 5 days and range of one to 44 days. As the ABC Study includes participants from across Australia, we analyzed the time from collection to receipt by state but did not find any substantial differences.

DNA extraction was successfully extracted from both the FOBT and ethanol samples. The DNA yield varied but reflected the amount of feces used; approximately 10 μg of DNA was extracted from 10 × 3 mm punches of the FOBT cards or 500 μL of the resuspended ethanol sample. DNA extracted from the ethanol samples was predominantly of high molecular weight (10 Kb). How well the sample was resuspended in ethanol by the participant did not influence DNA yield or quality (see Supplementary Table S1). The DNA extracted from FOBT cards had a lower molecular weight (ranging from 3 to 8 Kb). The DNA extracted from ethanol was very similar to DNA extracted from the control sample (a fecal sample frozen at −20°C within 1 hour of collection).

Of the subset of DNA samples that underwent metagenomic analysis (nine DNA samples, two from FOBT and one ethanol sample, from three participants). A low proportion of reads mapped to human or other reference sequences, indicating contamination (maximum = 0.3%, mean = 0.06%, across all samples), and the percentage of mapped versus unmapped reads was similar across collection methods.

**Figure 1.**
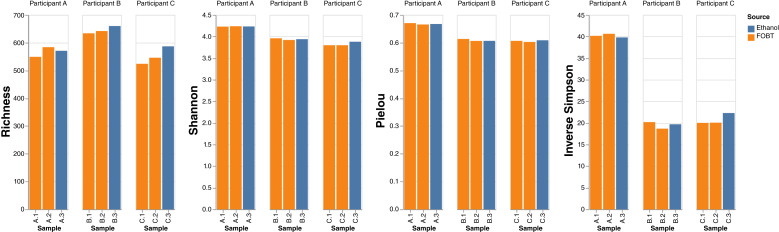
Per sample α-diversity measures from metagenomic analysis of whole-genome sequencing of DNA extracted from FOBT and ethanol for three ABC participants. Richness = number of species observed. Two FOBT samples were collected from each participant, denoted by orange. One ethanol sample was collected from each participant, denoted by blue.

The results of the inverse Simpson index, a measure of sample microbial diversity, were broadly similar for within-participant samples (Fig. 1). The results of the Pielou index, measuring evenness of species abundance, and the Shannon index, which considers species richness and evenness, were also similar within participant samples and broadly similar across participants. The average number of species observed was 580 (range, 524–642) for FOBT samples and 606 (range, 571–660) for ethanol samples.

**Figure 2.**
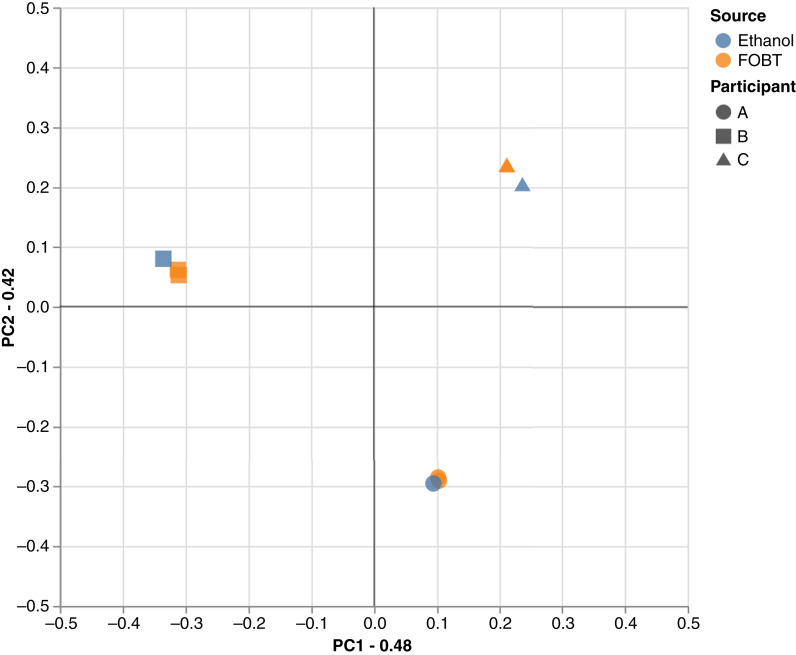
Principal coordinate analysis scatterplot displaying between sample diversity based on Bray–Curtis distance calculated at species level. The axis labels display the eigenvalues of the first two principal components (PC). Participants are denoted by shape, and sample source by color.

**Figure 3.**
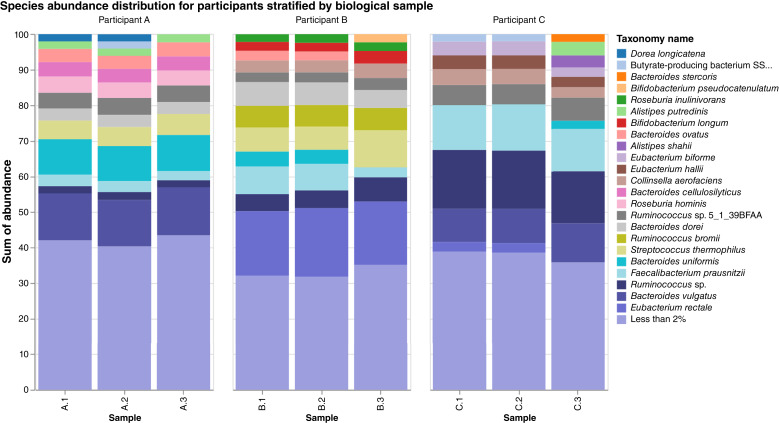
Relative species abundance distribution by participant and sample.

The principal coordinate analysis plot of the Bray–Curtis distance matrix demonstrated that the samples clustered by participant (Fig. 2). Within participants, though largely consistent between sampling methods, there may be a slightly greater dissimilarity between different sampling methods (FOBT vs. ethanol) than between different samples collected via the same method (FOBT1 vs. FOBT2). The species abundance distribution for participants stratified by sample also found largely similar relative abundance distribution within participants (Fig. 3).

### Extension to the larger ABC Study

Based on results of the pilot study, the decision was made to extend the invitation to complete all fecal sampling study components to the entire eligible ABC Study cohort. Over the period from 2020 to 2024, a total of 53,851 participants were invited to provide fecal samples via FOBT and ethanol, as well as to complete a dosQ and a FFQ. Of these, 29,158 (54.1%) opted-in to participate. Of the participants who opted-in, 22,670 (77.8%) provided a fecal sample of any kind, most of them providing both FOBT and ethanol samples (*n* = 22,250; 76.3%), and 22,561 (77.4%) completed the dosQ; a FFQ was completed by almost all (*n* = 27,008; 92.6%) participants who opted-in.

## Discussion

The pilot study demonstrated that asking participants to provide a fecal sample using a tube containing ethanol as well as on an FOBT card did not appreciably influence response to completing all components. Nor did the additional requirement to complete a FFQ, which is relatively long and complex, seem to reduce the likelihood of a participant completing all components. Nearly half (49%) of the 1,093 ABC Study participants invited provided a fecal sample. Adequate DNA was able to be extracted from the subset of samples selected for analysis, and the results of sequencing suggest that the methods of sample collection, transport, and storage utilized in the study are fully compatible with metagenomic analysis.

The feedback we received from nonresponders and noncompleters reinforced the need to communicate reminders to participants via multiple means of communication. In expanding fecal sample collection to the wider cohort, more reminders for both the sample provision and questionnaire completion were included, using a wider variety of reminder methods, including SMS and phone. In response to feedback from a subset of participants, we also updated sampling instructions to include the note “Ideally, collect the sample for the cards and the tube at the same time.” In the dosQ time/date question, we have now included a “do not know” option and a sentence to encourage participants to give their best estimate. Following these adjustments, the proportion of invited participants completing all components was 41% (76% of those who opted-in) following the extension of the fecal sample collection to the entire ABC Study cohort.

Previous studies have demonstrated the feasibility of home-based fecal sample self-collection for microbiome analysis (20, 21). FOBT-based microbiome analysis may have benefits for large prospective cohort studies, as these cards are relatively easy to transport, store, and collect. They have been utilized at scale in colorectal cancer screening programs and may be more stable at ambient temperatures than other methods (9). One study reported that FOBT-collected microbiome samples may be stable at room temperatures for at least 3 years (22). Although metagenomic and metabolomic analyses can be undertaken using samples collected from both FOBT and 95% ethanol, samples collected using FOBT cards have been reported to perform better for metagenomic analysis, whereas samples collected in 95% ethanol may be better for metabolomic analyses (5, 8, 11). Thus it may be optimal for studies to collect both ethanol and FOBT samples if both metagenomic and metabolomic analyses are planned.

The majority of published microbiome studies to date have utilized some form of stool sampling, as the intestine is the site of greatest colonization and hosts many of the most thoroughly studied microorganisms (23). The greatest effect of variation in gut flora is at the local level, and it follows that colorectal cancer is the malignancy most robustly associated with microbiome alterations (24–26), although other microbiome–cancer risk associations have been reported (27–29). It is anticipated that larger pooled gut microbiome studies with longer follow-up will be better able to detect associations with distal cancers, as variations in the gut microbiome may also have systemic effects via metabolic and other pathways (30, 31).

Most published studies investigating the microbiome and cancer risk have been small, cross-sectional, and only collected samples on a single occasion. Larger studies with repeated sample collection and a prospective design are currently being established to help elucidate the temporal associations between the composition of the gut microbiome and cancer, as well as other diseases. These studies may be better able to consider important host factors such as age, sex, antibiotic use, smoking, alcohol consumption, diet, obesity, physical activity, and genetic polymorphisms (2, 32). Conducting microbiome studies in diverse geographic settings is also important; large geographical differences in microbiome profile have been reported, even between “Western” populations from Europe and the United States (33), and detectable geolocation signals may even exist at an intracity level (34).

The findings of the pilot study lent confidence to plans to extend the scope of this work and invite all ABC Study participants to provide a fecal sample both on an FOBT card and in an ethanol tube, as well as completing both a dosQ and a FFQ. The pilot suggested that we could request that participants complete multiple study components without appreciably reducing the proportion of participants completing all components. Extending the pilot to the larger cohort will facilitate future metagenomic, metabolomic, and other host- and microbiome-related analyses in this setting, or as part of an extended pooled cohort. Repeat sampling in future planned follow-up will allow assessment of alterations in microbiome profile and the potential associations of these alterations with disease states. Further studies and collaboration are required to define standard protocols that will allow reproducibility across microbiome analyses and pooling of diverse cohorts (35, 36).

## Supplementary Material

Supplementary Table S1 Table S1. Characteristics of FOBT and ethanol faecal samples that underwent trial DNA extraction

Supplementary Figure S1 Figure S1. Initial invitation to pilot

Supplementary Figure S2 Figure S2. Reminder email to non-respondents

Supplementary Figure S3 Figure S3. Reminder to send faecal samples

Supplementary Figure S4 Figure S4. Faecal sample collection instructions

Supplementary Figure S5 Figure S5. Photograph of sample collection kit for groups C&D

Supplementary Figure S6 Figure S6. Cover letter accompanying sample collection kit

## Data Availability

The data underlying this article cannot be shared publicly due to compliance with participant consent and existing ethics approvals. They can be made available on reasonable request, with appropriate ethics approval, to PEDIGREE@cancervic.org.au.
