# Supplementary material for: Fecal Sample Collection for Gut Microbiome Research in a Prospective Cohort: A Pilot Study within the Australian Breakthrough Cancer Study
Source: Cancer Res Commun. 2026 Jan 9;6(1):70–6. doi: 10.1158/2767-9764.CRC-25-0445 (PMC12784011; doi:10.1158/2767-9764.CRC-25-0445)
Supplement: Supplementary Table S1 — Table S1. Characteristics of FOBT and ethanol faecal samples that underwent trial DNA extraction [file crc-25-0445_supplementary_table_s1_suppst1.docx]

**Supplementary Table S1: Characteristics of FOBT and ethanol faecal samples that underwent trial DNA extraction**

| **Sample** | **Sex** | **Age at baseline** | **State** | **Time in transit (days)** | **Comments** |
| --- | --- | --- | --- | --- | --- |
| 1 | Male | 58 | VIC | 3 | FOBT: NC  Ethanol: Sample resuspended in ethanol |
| 2 | Female | 59 | VIC | 3 | FOBT: Small faecal sample deposited on FOBT.  Ethanol: Stool not resuspended in ethanol, transferred clump |
| 3 | Male | 56 | WA | 7 | FOBT: NC  Ethanol: Sample resuspended in ethanol |
| 4 | Female | 62 | QLD | 10 | FOBT: NC  Ethanol: Sample not resuspended in ethanol, transferred clump |
| **Control** |  |  | VIC | 0 | No FOBT or ethanol. Aliquoted and snap frozen at -20⁰_C_ on day of collection |

*VIC, Victoria; WA, Western Australia; QLD, Queensland; NC, no comment.
