# Supplementary figures and images for: Fecal Sample Collection for Gut Microbiome Research in a Prospective Cohort: A Pilot Study within the Australian Breakthrough Cancer Study
Source: Cancer Res Commun. 2026 Jan 9;6(1):70–6. doi: 10.1158/2767-9764.CRC-25-0445 (PMC12784011; doi:10.1158/2767-9764.CRC-25-0445)

**Supplementary Figure S1: Initial invitation to pilot**

**
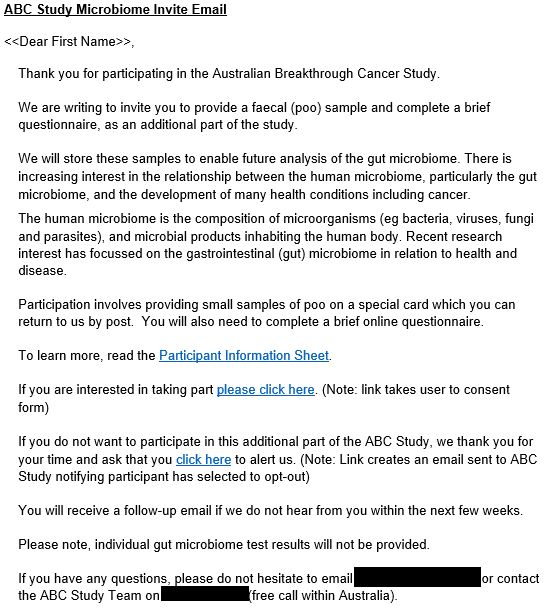
**

Supplement: Supplementary Figure S1 — Figure S1. Initial invitation to pilot [file crc-25-0445_supplementary_figure_s1_suppsf1.docx]

**Supplementary Figure S2: Reminder email to non-respondents**

**
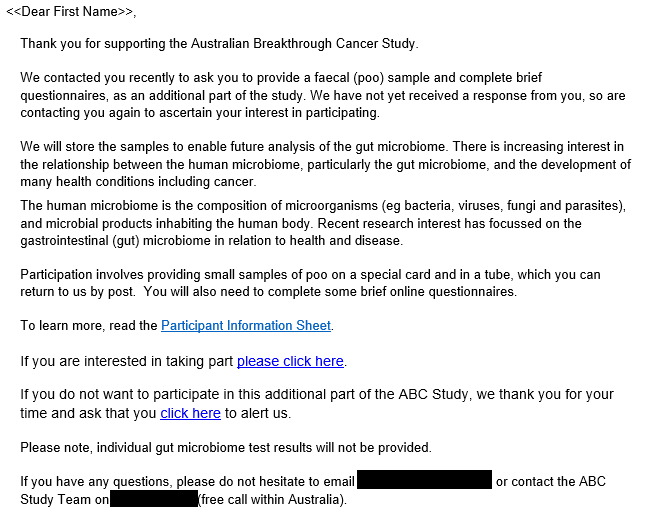
**

Supplement: Supplementary Figure S2 — Figure S2. Reminder email to non-respondents [file crc-25-0445_supplementary_figure_s2_suppsf2.docx]

**Supplementary Figure S3: Reminder to send faecal samples**


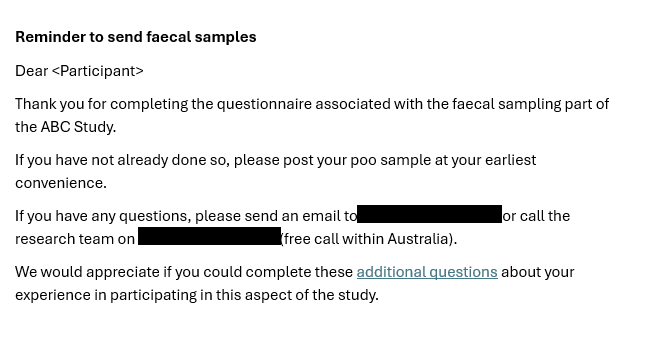

Supplement: Supplementary Figure S3 — Figure S3. Reminder to send faecal samples [file crc-25-0445_supplementary_figure_s3_suppsf3.docx]

**Supplementary Figure S4: Faecal sample collection instructions**


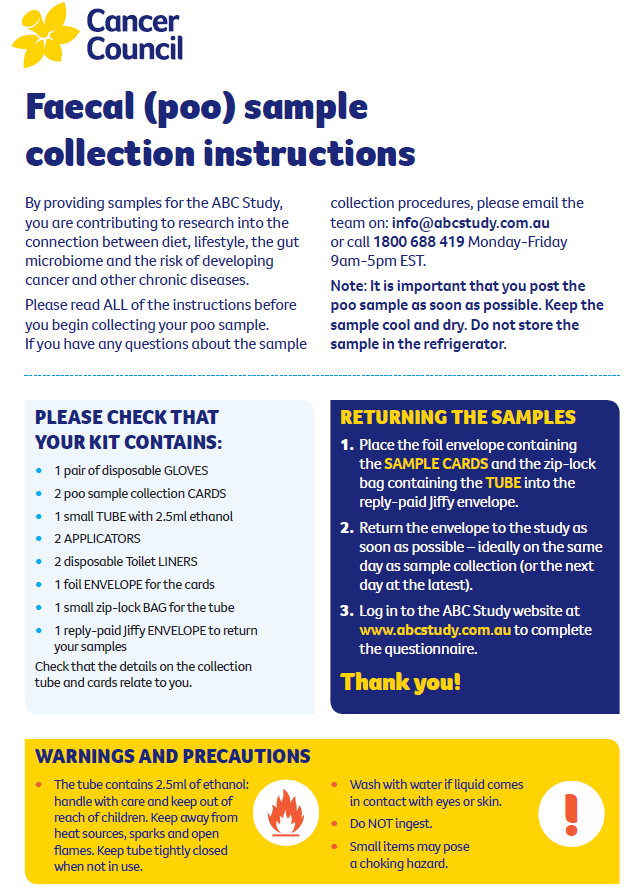


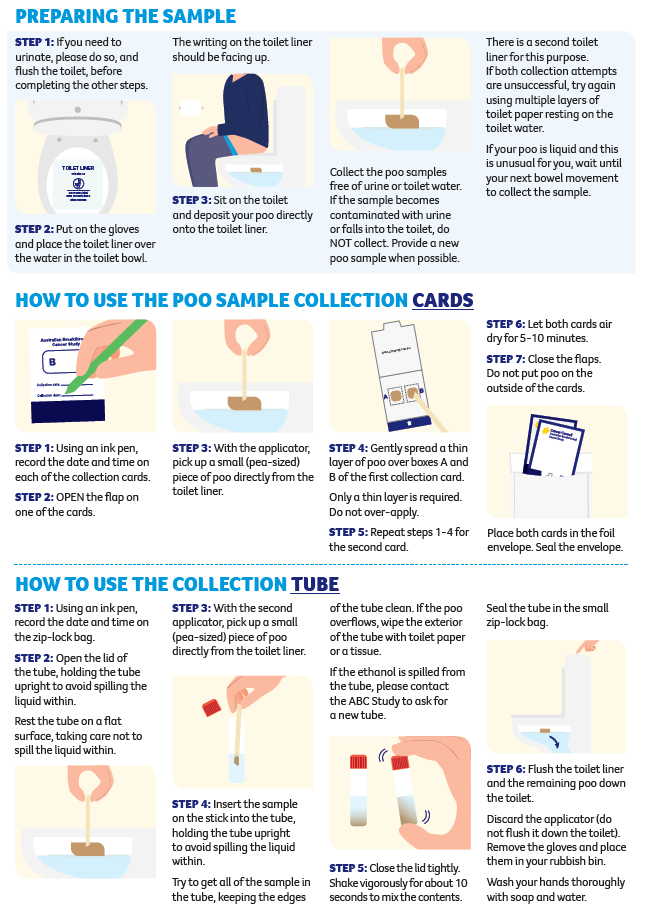

Supplement: Supplementary Figure S4 — Figure S4. Faecal sample collection instructions [file crc-25-0445_supplementary_figure_s4_suppsf4.docx]
