# Supplementary material for: Fecal Sample Collection for Gut Microbiome Research in a Prospective Cohort: A Pilot Study within the Australian Breakthrough Cancer Study
Source: Cancer Res Commun. 2026 Jan 9;6(1):70–6. doi: 10.1158/2767-9764.CRC-25-0445 (PMC12784011; doi:10.1158/2767-9764.CRC-25-0445)
Supplement: Supplementary Figure S5 — Figure S5. Photograph of sample collection kit for groups C&D [file crc-25-0445_supplementary_figure_s5_suppsf5.docx]

**Supplementary Figure S5: Photograph of sample collection kit for groups C&D**

**
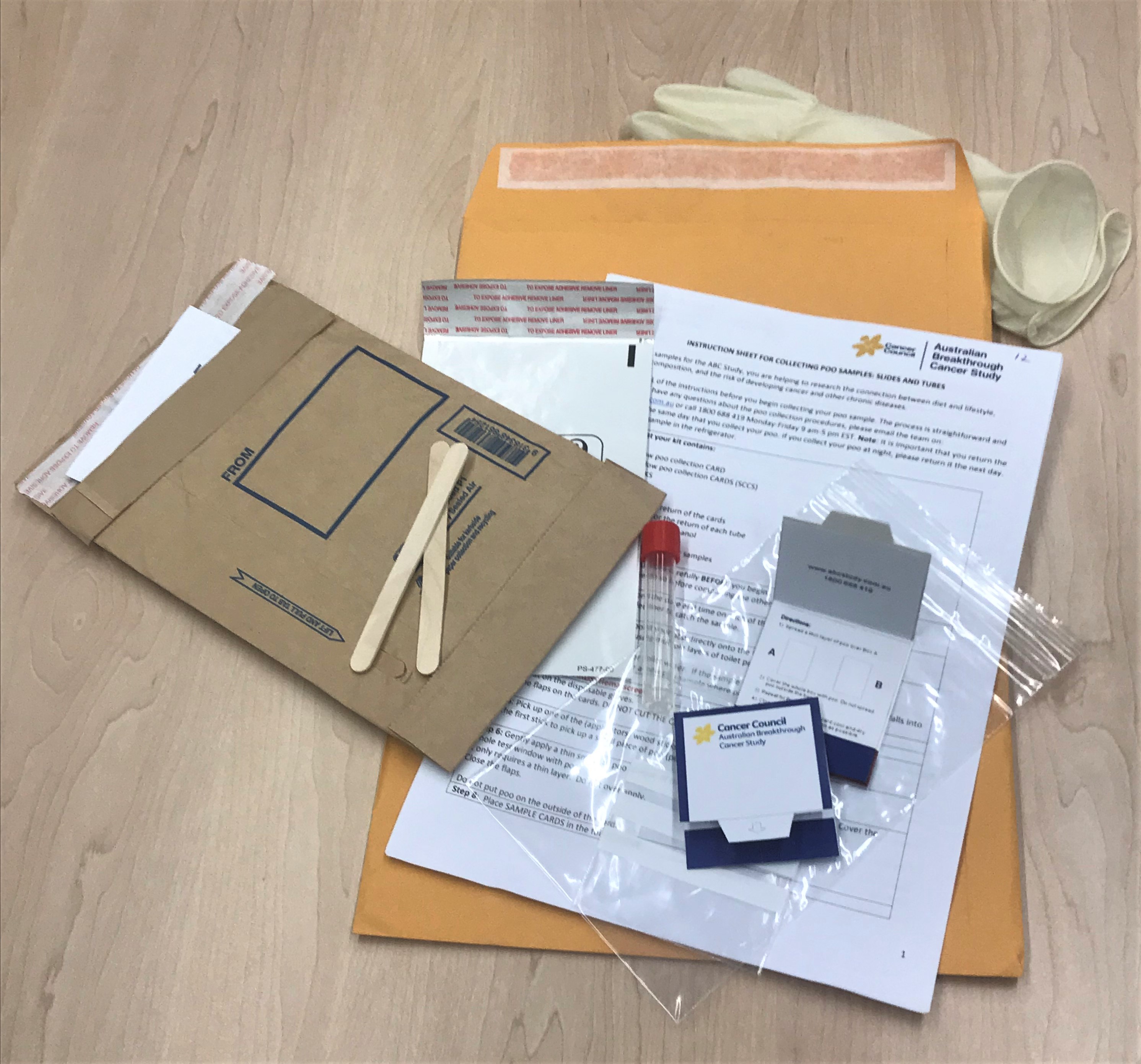
**

NB. Contents for participants in groups A&B were similar, except they did not receive the ethanol containing tube or zip lock bag.
