# Supplementary material for: Fecal Sample Collection for Gut Microbiome Research in a Prospective Cohort: A Pilot Study within the Australian Breakthrough Cancer Study
Source: Cancer Res Commun. 2026 Jan 9;6(1):70–6. doi: 10.1158/2767-9764.CRC-25-0445 (PMC12784011; doi:10.1158/2767-9764.CRC-25-0445)
Supplement: Supplementary Figure S6 — Figure S6. Cover letter accompanying sample collection kit [file crc-25-0445_supplementary_figure_s6_suppsf6.docx]

**Supplementary Figure S6: Cover letter accompanying sample collection kit**
